# Redesigning therapies for pantothenate kinase–associated neurodegeneration

**DOI:** 10.1016/j.jbc.2022.101577

**Published:** 2022-01-15

**Authors:** Muhammad I. Munshi, Sarah J. Yao, Choukri Ben Mamoun

**Affiliations:** Section of Infectious Diseases, Department of Internal Medicine and Department of Microbial Pathogenesis, Yale University School of Medicine, New Haven, Connecticut, USA

**Keywords:** neurodegeneration, pantothenate kinase–associated neurodegeneration, coenzyme A, iron, mitochondria, gene therapy, drug design, Alzheimer's disease, Parkinson's disease, CNS, central nervous system, CoA, coenzyme A, GP, globus pallidus, mtACP, mitochondrial acyl carrier protein, P-Pa, 4′-phosphopantothenate, P-PaSH, 4′-phosphopantetheine, PKAN, pantothenate kinase–associated neurodegeneration, SYN1, synapsin-1, TCA, tricarboxylic acid

## Abstract

Pantothenate kinase–associated neurodegeneration (PKAN) is an incurable rare genetic disorder of children and young adults caused by mutations in the *PANK2* gene, which encodes an enzyme critical for the biosynthesis of coenzyme A. Although PKAN affects only a small number of patients, it shares several hallmarks of more common neurodegenerative diseases of older adults such as Alzheimer's disease and Parkinson's disease. Advances in etiological understanding and treatment of PKAN could therefore have implications for our understanding of more common diseases and may shed new lights on the physiological importance of coenzyme A, a cofactor critical for the operation of various cellular metabolic processes. The large body of knowledge that accumulated over the years around PKAN pathology, including but not limited to studies of various PKAN models and therapies, has contributed not only to progress in our understanding of the disease but also, importantly, to the crystallization of key questions that guide future investigations of the disease. In this review, we will summarize this knowledge and demonstrate how it forms the backdrop to new avenues of research.

Pantothenate kinase–associated neurodegeneration (PKAN) is a rare autosomal recessive disorder that largely affects children and young adults (1). The disease results from mutations in the pantothenate kinase gene (*PANK2*), which encodes the first enzyme in the biosynthesis of coenzyme A (CoA) from pantothenic acid (vitamin b5). The CoA biosynthesis pathway has been shown to play a critical role in cellular metabolism with reports suggesting that between 4% and 9% of all cellular metabolic activities rely on CoA as a cofactor, including the tricarboxylic acid (TCA) cycle, fatty acid regulation, amino acid synthesis, and lipid metabolism (2).

The clinical presentation of PKAN can be broadly divided into two forms: early-onset PKAN and late-onset PKAN (3). Roughly 50% of all PKAN patients suffer from early-onset PKAN, presenting symptoms before age 10 (typically at age 3) in the form of extrapyramidal disorders like dystonia, dysarthria, and rigidity (3). These symptoms worsen progressively over the patient’s life span leading to loss of ambulatory capacity as well as severe and painful dystonia (3). Curiously, even though the genetic mutations in the *PANK2* gene might be predicted to affect all cells, the pathology of PKAN appears to be highly focal, centered in neurons of the globus pallidus (GP) and in retinal rods, which have high energy and myelin maintenance demand (4). Furthermore, in some patients with PKAN, iron accumulation in the GP and to a lesser extent in the substantia nigra, gives rise to a distinctive “eye of the tiger” signature on an MRI scan (Fig. 1).

**Figure 1:**
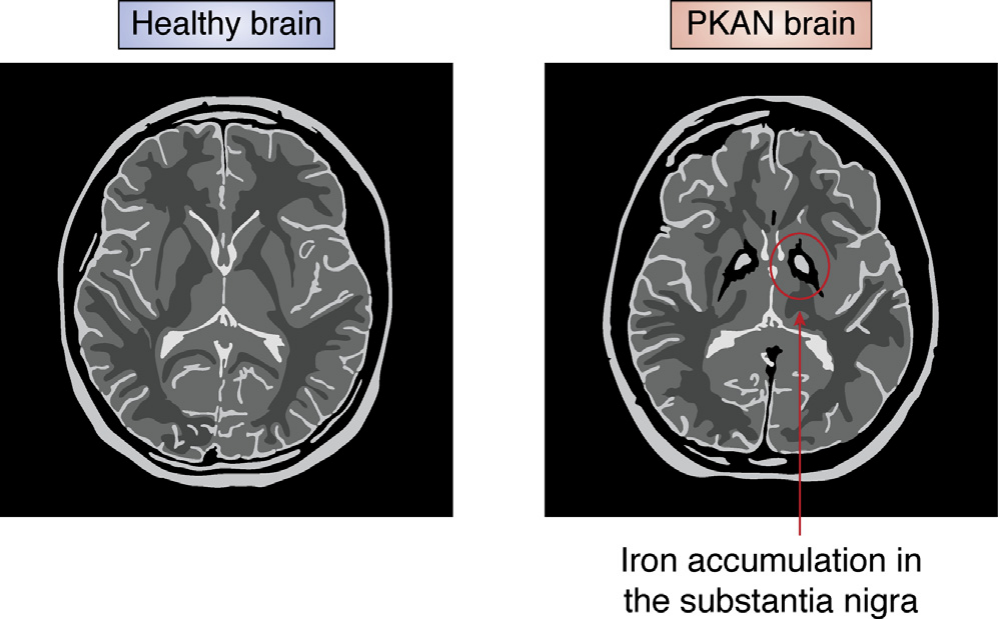
**Cartoon representation of T2-weighted MRI of control patient (*left*) and PKAN patient (*right*).** The *bright spots* in the substantia nigra are iron deposits that give rise to the characteristic of the “eye of the tiger” MRI seen in PKAN patients. PKAN, pantothenate kinase–associated neurodegeneration.

Later-onset PKAN, in contrast, is associated with a varied spectrum of symptoms that tend to present first in the patients' teenage years (typically at age 18) (3). The most common symptoms include speech disorders and psychiatric symptoms, but more severe motor symptoms, such as Parkinsonism and dystonia, can develop as patients age (3).

No disease-altering treatment is currently available for PKAN. Patients with typical disease progression experience a sharp decline and death in their teenage years (5). Moreover, extrapyramidal symptoms associated with PKAN are also observed in more common neurodegenerative diseases like Parkinson's disease and Alzheimer’s disease (6, 7).

Further understanding of the molecular and biochemical mechanisms underlying PKAN could therefore shed new lights on the metabolic defects that serve as precursors for other more common neurodegenerative diseases. Here, we outline key findings from model organisms and cell lines to evaluate proposed disease etiologies (Table 1 and Fig. 2). In light of these findings, we highlight the strengths and weaknesses of various therapeutic approaches.

**Table 1:** Molecular perturbations and neurodegenerative phenotypes associated with PANK-associated CoA deficiency

Model for reduction in CoA levels/mutation type	Species	Reference	Neurodegenerative phenotype	Molecular perturbation	Complementation	Relevant treatment/rescue strategy for neurodegeneration	Mammalian model limitations
*Pank2*^*−/−*^ (homozygous null)	*Mus musculus*	(13, 24)	Retinal degeneration, growth reduction	Decreased expression of enzymes in the CoA pathway; increased iron accumulation; reduced complex I, pyruvate dehydrogenase, and mitochondrial aconitase activities	N/A	4′-Phosphopantetheine supplementation	Failed to exhibit PKAN-related movement disorder, even when followed over 16 months of age
*Pank2*^*−/−*^ with ketogenic diet (homozygous null)	*Mus musculus*	(15)	Severe dystonia and locomotor disorders, alteration of muscle morphology, life span reduction	Mitochondrial degeneration, reduced oxygen consumption rate (OCR), accumulation of ubiquitinated proteins	N/A	Pantethine administration	No iron accumulation; dietary shift not observed in patients
*Pank1*^*−/−*^*Syn*^*Cre/+*^*Pank2*^*fl/fl*^ (systemic KO of *PANK1* and neuronal-specific KO of *PANK2*)	*Mus musculus*	(11)	Decreased locomotor function, abnormal gait, weight loss, and shortened life span	Reduced CoA levels in forebrain and midbrain, 12% iron reduction in the brain	N/A	N/A	Iron levels are lower in the brain of mutants relative to WTs, contrasting with PKAN patients; CoA deficiency is more severe than in PKAN patients; mice die by day 17 of life
*Syn*^*Cre/+*^*Pank1*^*fl/fl*^*Pank2*^*fl/fl*^ (neuronal-specific KO of *PANK1* & *PANK2*)	*Mus musculus*	(14)	Weight loss, severe locomotor impairment, and reduced life span	Reduced CoA levels in the brain	N/A	Pank1 and Pank3 activators (*i.e.*, PZ-2891)	N/A
Syn-Nudt7cyt (neuron-specific overexpression of cytosolic Nudt7)	*Mus musculus*	(34)	Decreased motor coordination	15% reduction in CoA levels in brain because of Nudt7-mediated CoA degradation	N/A	N/A	N/A
*Pank1*^*−/−*^*Pank3*^*−/−*^ (homozygous null)	*Mus musculus*	(60)	Embryonic lethal	N/A	N/A	N/A	N/A
*Pank2*^*−/−*^*Pank3*^*−/−*^ (homozygous null)	*Mus musculus*	(60)	Embryonic lethal	N/A	N/A	N/A	N/A
*dPANK/fbl* (hypomorph)	*Drosophila melanogaster*	(21, 23, 26)	Decreased locomotor function; increased apoptosis in larval brains; decreased life span	Reduced histone and tubulin acetylation; impaired DNA damage response; increased cysteine hypersensitivity	*fbl* mitochondrial splice variant and hPank2	Pantethine supplementation; HDAC inhibitors	N/A
dPANK2 knockdown	*Danio rerio*	(20, 25)	Impaired neuronal development	Reduced expression of neurog1 and wnt1, which are key transcription factors in neurogenesis	Overexpression of hPANK2	CoA, pantethine	N/A
*Cab1ts* (temperature-sensitive hypomorph)	*Saccharomyces cerevisiae*	(22, 43, 44)	N/A	Mitochondrial dysfunction, iron dyshomeostasis, increased lipid peroxidation, and glycolytic shift	hPANK2 cDNA with mitochondrial-targeting motif failed to complement Cab1ts	Nalidixic acid Pantothenic acid 5,7 dichloro-8 hydroxyquinoline (CQ_cl_)	N/A

Abbreviations: cDNA, complementary DNA; HDAC, histone deacetylase; hPANK2, human Pank2; N/A, not available.

**Figure 2:**
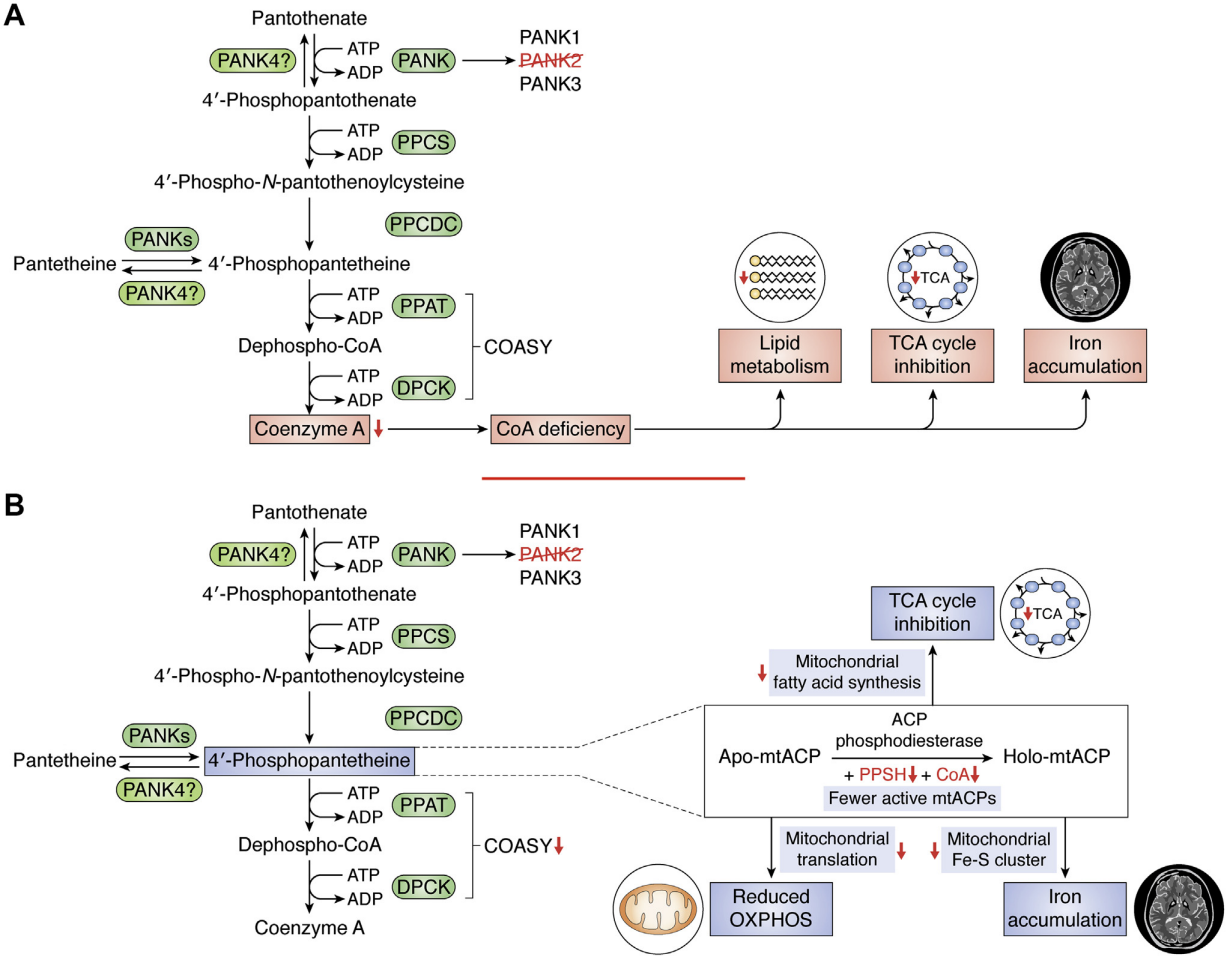
**Etiology of PKAN.***A*, model of PKAN pathogenesis based on whole-cell CoA deficiency. *B*, model of PKAN pathogenesis based on deficiency in mitochondrial acyl carrier proteins (mtACPs). DPCK, dephosphocoenzyme A kinase; PKAN, pantothenate kinase–associated neurodegeneration; PPAT, phosphopantetheine adenylyl transferase; PPCDC, phosphopanthenoylcysteine decarboxylase; PPCS, phosphopantothenoylcysteine synthetase.

## Etiology of PKAN

### CoA deficiency

Although studies conducted so far in various models have led to a significant improvement of our knowledge about the etiology of PKAN, there remains large gaps in our understanding of the molecular and cellular mechanisms underlying its pathology. Most hypotheses of pathogenesis focus on the reduction in CoA levels in key brain regions and particularly in the GP. One proposed mechanism of pathogenesis is that a mutant PANK2 enzyme with reduced activity leads to lower levels of cellular CoA with subsequent impacts on other cellular metabolic machineries including the TCA cycle and lipid metabolism (Fig. 2*A*). Since CoA is involved in a vast array of cellular metabolic processes, low levels of this cofactor may be responsible for the PKAN defects seen in PANK2-deficient cells (8). The finding that CoA levels in fibroblasts from some PKAN patients are similar to those of healthy controls has cast doubt on the direct link between CoA levels and PKAN pathology (9, 10). However, there could be other possible explanations for observed normal whole cell CoA levels in PKAN cells. First, measurement of the labile unacylated CoA pool is technically challenging, and there has been some controversy over the accuracy of these measurements (11). A second possible explanation is that the CoA deficiency might be specific to GP neurons, cells with the highest energy and myelin maintenance demand (4). A third possible explanation for this observation is that a critical *subcellular* pool of CoA might be depleted in PKAN (12), and differences in CoA levels on the whole cell/tissue level are unchanged. This hypothesis is supported by the role of mitochondrial acyl carrier proteins (mtACPs), in directly “depleting” CoA from the mitochondrial compartment (Fig. 2*B*) (12). In most metabolic reactions that are CoA dependent, CoA is acylated, and then an acyl carrier protein transfers the acyl moiety to another molecule, regenerating CoA in the process (13). However, some specific proteins, like cytosolic fatty acid synthase and mtACPs, the full 4′-phosphopantetheine (P-PaSH) moiety from CoA forms a 4′phosphopanthetinylated protein and releases an adenosine 3′,5′-bisphosphate (12). Removal of the entire P-PaSH moiety prevents regeneration of CoA, effectively “depleting” the pool of CoA available (12). It has been suggested that the CoA required for this process could be part of a specific subcellular pool that requires PANK2 to be generated. If this specific pool is depleted, then it is possible that the cell experiences a shortage in activated mtACPs without showing an overall shortage of CoA at the cellular level. This hypothesis is compelling because mtACPs play a key role in various metabolic pathways known to be affected in PKAN, including the electron transport chain, fatty acid synthesis, and iron–sulfur cluster biogenesis (13). To activate apo-mtACP and form holo-mtACP, a full 4′-phosphopantethine moiety is transferred from CoA. Holo-mtACP is then used most prominently in complex I of the electron transport complex, specifically, the NDUFAB1 subunit in humans (12). Moreover, holo-mtACP is also required for the formation of acyl-ACPs like octanoyl-ACP, a precursor for lipoic acid, which plays a key role in the activation of pyruvate dehydrogenase (12). Acyl-ACPs have also been linked to iron–sulfur cluster biogenesis, which are critical to both mitochondrial function and iron homeostasis (12). Defects in mtACP production, as a result of insufficient supplies of P-PaSH, in PKAN patients could therefore explain many of the most common defects seen in patients as well as in mammalian and eukaryotic models of PKAN.

### Iron accumulation

Although iron accumulation is perhaps the most striking feature of PKAN, it is widely accepted that the pathogenesis of PKAN in humans occurs because of a metabolic imbalance rather than proceeding from iron accumulation. This hypothesis is supported in part by data from clinical trials using iron chelators, which failed to produce significant improvement in symptoms (5, 14), as well as data from mouse models (11, 14, 15). Nevertheless, even if iron accumulation is not a part of the initial metabolic cascade that causes PKAN, it could still be a major factor in driving disease progression, and iron accumulation is an important factor in other neurodegenerative diseases (6, 7). It is therefore critical to understand the molecular mechanism underlying iron accumulation in PKAN. Such knowledge could help elucidate the metabolic and cellular impacts of CoA deficiency in the evolution of the disease and would guide the design of more effective therapeutic strategies to treat and reverse disease progression.

Several hypotheses for iron imbalance in PKAN have been proposed. One hypothesis links iron imbalance to cysteine accumulation as a result of a defective PANK2 activity (16). Following phosphorylation of pantothenic acid, cysteine is conjugated to the product 4′-phosphopantothenate (P-Pa) to form 4′-phosphopantothenoylcysteine, a reaction catalyzed by phosphopantothenoylcysteine synthetase. Reduced PANK2 activity could lead to accumulation of cysteine and cysteine-related compounds such as *N*-pantothenoylcysteine. Because of its iron-chelating activity, excess cysteine levels could lead to the formation of deposits of nonbioavailable iron seen in “eye of the tiger” MRIs of PKAN patients. Moreover, rapid autoxidation of cysteine in the presence of iron could generate free radicals resulting in oxidative injury to cells (1). Finally, enhanced lipid peroxidation in the presence of iron, a possible secondary mechanism of pathogenesis in PKAN, is also enhanced by the presence of cysteine, potentially damaging cell membranes and leading to apoptosis (1). Therefore, cysteine toxicity could be a cause of iron dyshomeostasis in PKAN and a plausible cause for increased oxidative stress and neurodegeneration. Iron can also accumulate in the form of lipofuscin granules with very high iron content (roughly 5%) (17). Lipofuscin is composed of oxidized aggregate of covalently cross-linked lipids and proteins. It is not easily degraded by lysosomes or proteasomes and was shown to be significantly increased in PKAN fibroblasts (18). Moreover, their high iron content could drive iron-dependent apoptosis and contribute to neurodegeneration. The mechanism by which lipofuscin elevation occurs remains unknown. Finally, iron dyshomeostasis in PKAN could be caused by impaired iron–sulfur cluster and heme biosynthesis (13). Iron–sulfur cluster plays a critical role in the function of mitochondrial enzymes, and heme is a porphyrin group involved in carrying oxygen. If both processes are downregulated, unused iron might accumulate in the mitochondria causing damage *via* generation of free radicals (13). Importantly, previous work has shown that iron dyshomeostasis in the mitochondria can lead to cytosolic iron deficiencies, which trigger a subsequent increase in iron uptake, thereby compounding the mitochondrial dyshomeostasis (19).

Overall, answering the question of how iron accumulation occurs in PKAN cells is vital both for determining a more complete etiology of PKAN and for its applications to other neurodegenerative diseases like Parkinson's disease and Alzheimer’s disease. Further studies in various models of PKAN could help to identify specific biomarkers of iron accumulation and guide further research into the specific mechanisms that drive iron dyshomeostasis.

## Models of PKAN

In recent years, several animal models of PKAN have been developed, but translation of this knowledge to humans is hampered by physiological and metabolic differences between model organisms and humans (11, 13, 14, 15, 20, 21, 22, 23, 24, 25, 26). One key difference is that while all the species used share a homologous pantothenate kinase gene, the number of different orthologs and subcellular localization of the encoded proteins often vary. The human genome encodes four PANK-like genes, three of which *PANK1*, *PANK2*, and *PANK3*, encode active dimeric pantothenate kinases with different subcellular localizations (8). PANK2 harbors a mitochondrial localization signal and has been localized largely to the mitochondria, whereas PANK1 and PANK3 are generally found in the nucleus and cytosol (8). *PANK4* encodes a protein that lacks the residues required for kinase activity but instead carries a phosphatase domain (DUF89) and has been shown to catalyze the dephosphorylation of P-PaSH and its derivatives as well as P-Pa (27, 28). Genome-wide transcriptomic analyses have shed new light on the tissue distribution of human PANKs. *PANK1* is primarily expressed in the liver, kidney, brain, and small intestine but expressed at low levels in other tissues like the liver and skeletal muscle (reads per kilobase of transcript, per million mapped reads <1) (29). *PANK2*, on the other hand, is expressed at moderate levels in most tissues but is highly expressed in the brain, thymus, and prostate (22). *PANK3* is expressed highly in the brain, heart, thymus, and skeletal muscle, with moderate expression levels in other tissues (reads per kilobase of transcript, per million mapped reads >1) (22). Finally, *PANK4* is highly expressed in the brain, prostate, small intestine, and adrenal gland (22). Similar to humans, mice express four *Pank* genes with varying tissue distribution. *Pank1* is highly expressed in the liver, adrenal gland, reproductive tissues, and brain of adult mice (30). *Pank2* is highly expressed in the limbs, brain, and central nervous system (CNS) of adult mice (30). *Pank3* is highly expressed in the brain, CNS, and adipose tissue. Finally, *Pank4* is highly expressed in the brain, thymus, limbs, and CNS (30). Little is known about the impact of the relative expression of each enzyme on CoA levels in healthy and Pank2-deficient tissues; however, further understanding of this differential expression could have an impact on therapeutic strategies for PKAN.

Adding to the complexity of the divergent subcellular localization and tissue distribution of the different isoforms is the feedback regulation pantothenate kinases experience by CoA or its acyl derivatives. It was proposed that the distinct subcellular localizations enable Panks to act as metabolic “sensors” that modulate the rate of CoA synthesis to align with the metabolic requirements of the cytosol (where CoA concentrations range from 0.02 and 0.14 mM) or of the mitochondria (where sequestered CoA levels range from 2.2 to over 5 mM) (31). Pank2, which is highly expressed in the brain (32), is the most sensitive Pank to negative feedback by acetyl-CoA, with an IC_50_ of ∼0.1 μM (33). That, combined with the compartmentalization of Acetyl-CoA, lead to a scenario where the effects of fluctuations in the levels of acetyl-CoA are not diluted out (as they are in the cytoplasm), and Pank2 is more likely to directly sense and respond to a significant proportion of the mitochondrial CoA pool. Consequently, mutations that compromise Pank2 function are expected just not only to reduce CoA levels and levels of its downstream products but also to alleviate the inhibition by acetyl-CoA. Mitigation of negative regulation may partially explain why several point mutations in human Pank2 either maintain or only partially reduce enzyme activity.

Unlike higher eukaryotes, which express more than one PANK enzyme, fungi encode a single PANK ortholog (22). The *Saccharomyces cerevisiae* Pank ortholog, Cab1, is particularly interesting because of its essential role in cell viability as well as for the presence of mutants that display cell biological defects similar to those seen in PKAN cells.

### Murine models of PKAN

**Figure 3:**
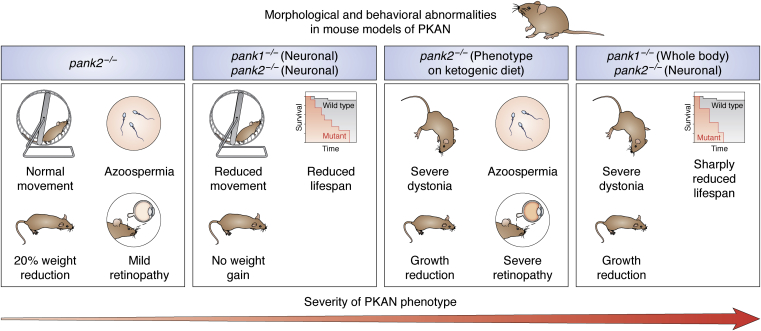
**Abnormalities of mouse models of PKAN.** Four PKAN mouse models with altered movement, size, life span, fertility, and vision are described. Although the PKAN phenotype is least severe in *Pank2*^*−/−*^ mice, the model is considered ideal for studying cellular etiology of PKAN. *Pank1*^*−/P*^*pank2*^*−/−*^ mice present more severe morphological and behavioral abnormalities, not all of which recapitulate the PKAN phenotype in humans. PKAN, pantothenate kinase–associated neurodegeneration.

Several mouse models have been used to study PKAN and are summarized in Table 1. Recent research has focused primarily on two models: mice with a whole-body knockout of *PANK2* and mice with targeted disruption of *PANK2* alone or in conjunction with *PANK1* in the brain and other organs (Table 1 and Fig. 3).

#### PANK2-deficient mice

The mouse model most relevant to PKAN is the whole-body knockout of *PANK2*. This model has been shown to exhibit a wide range of cellular defects associated with PKAN in the brain and eyes, making it an ideal model to study cellular etiology of PKAN. The model, however, does not exhibit significant morphological and motor function abnormalities (13). At the molecular level, *pank2*^*−/−*^ mice show localized defects in the GP but normal function elsewhere (13). Significant differences in the transcript levels of genes encoding key enzymes in the CoA pathway—phosphopantothenoylcysteine synthetase and CoA synthase—were found the in the GP of these mice, but normal function was observed in other parts of the brain. The GP is the focal point for iron deposits in the brains of human PKAN patients, and therefore, defects in the GP are hypothesized to be at the root of the extrapyramidal motor symptoms associated with PKAN. Pathological studies of the brains of PKAN patients align with the GP specificity seen in *pank2*^*−/−*^ mouse brain (34), which shows evidence of iron dyshomeostasis localized to the GP. Iron levels were increased by roughly 27% in the mitochondria and cytosol, and key enzymes that control cellular iron levels were significantly downregulated in the GP of the mouse brain (13).

Although *pank2*^*−/−*^ mice were not altered in their motor function and did not display signs of Parkinsonism, expression levels of dopamine receptors, DRD1 and DRD2, and tyrosine hydrolase were altered, suggesting that dopamine homeostasis might also be affected in these animals. Dopamine is a critical neurotransmitter involved in motor function and control, and lack of dopamine is a defining symptom of Parkinson’s disease (35). Dopamine dyshomeostasis could provide the critical link between lack of CoA availability and the extrapyramidal disorders commonly seen in PKAN patients. One hypothesis is that dopaminergic neurons might be more sensitive to the iron overload found in PKAN, thereby causing a dopamine deficiency, but this hypothesis has yet to be validated (13).

Overall, while the *pank2*^*−/−*^ model is useful because of its direct genetic link to PKAN, the mice do not display apparent morphological defects or movement disorder symptoms. While movement disorders, retinopathy, and azoospermia were observed when *pank2*^*−/−*^ mice were put on a ketogenic diet, these disorders are seen in human patients independent of the diet provided (15).

#### A mouse model of PKAN with dual disruption of *PANK1* and *PANK2* in the brain

One proposed reason for the lack of a severe phenotype in the *pank2*^*−/−*^ mice is that both PANK1 and PANK2 are important for brain development in mice and are expressed at high levels in the CNS, so deletion of *Pank2* could be compensated for by increased *Pank1* expression (11). Sharma *et al.* (14) reported a mouse model in which both *Pank1* and *Pank2* were deleted in the brain using the Cre recombinase expressed under the regulatory control of the synapsin-1 (SYN1) promoter. In this mice with selective neuronal *Pank1* and *Pank2* deficiency, pronounced morphological defects including weight loss and severe locomotor impairment were observed (14). In addition, this mouse was found to have reduced CoA levels in the brain. However, the effect of loss of both *Pank1* and *Pank2* on iron levels and dopamine homeostasis has not been reported. Although the phenotype reported in this transgenic mouse is more severe than what is seen in PKAN patients, this model reinforces an important association between neuronal CoA levels and motor function, a connection that was demonstrated earlier by the same group in a Syn-*NUDT7* mouse model (20, 21). The *pank2*^*−/−*^ mouse model may therefore offer an additional resource for further evaluation of clinical candidates for PKAN treatment.

#### *Pank2* disruption in the brain of mice with *Pank1* whole-body knockout

Another recent mouse model of PANK2 deficiency used a whole-body deletion of *PANK1* and a neuronal deletion of *PANK2* (11). The mice showed evidence of extrapyramidal disorders with a reduced median life span of 17 days postnatal (before the end of weaning) (14). These animals also showed several metabolic defects with more than 30% reduction in CoA levels in the brain and spinal cord and ∼50% reduction in succinyl-CoA levels (11). Consistent with the important role succinyl-CoA plays in the TCA cycle and heme biosynthesis, brain heme levels were also found to be reduced by ∼50% once movement disorders developed in the mice (11). Heme proteins play a key role in mitochondrial redox regulation, and alteration in hemoglobin levels has been observed in patients with Parkinson’s disease and Alzheimer’s disease (17, 36, 37). Interestingly, measurement of iron levels in mice 19 to 21 days postnatal showed a 12% reduction in forebrain iron levels in *pank1*^*−/−*^, *Syn*^*Cre*^ *pank2*^*−/−*^ knockout mice as compared to WT mice (11). This contrasts with the iron accumulation observed in the GP of human PKAN patients. The discrepancy could be due to the use of the whole forebrain for iron measurements, as models of *pank2*^*−/−*^ showed significant increases in iron levels in the GP only (13). However, if the measurements are unchanged, this might suggest that iron accumulation is developed later in the disease course, and it is merely an epiphenomenon rather than the major driver of disease progression. Further studies of the role of iron, heme, and hemoglobin levels, as well as dopamine homeostasis, in this model might shed further light on a possible mechanism of pathogenesis in PKAN, including the processes that precede iron accumulation.

However, one key weakness of these mouse models is that they target both *PANK1* and *PANK2*, whereas PKAN arises from disruption of only *PANK2* in humans. This might make the findings from these models more relevant to advancing our understanding of CoA deficiencies overall rather than understanding the etiology of PKAN specifically. Another difference between human and mouse Pank2 is that the latter has been localized primarily to the cytoplasm (33). This begs the question of whether this key difference undermines efforts aimed at obtaining a murine phenotype more representative of PKAN, even when utilizing different combinations of isoform knockouts. Is a mitochondrial targeted Pank a prerequisite for establishing a PKAN phenotype? Is the intimate association between a mitochondrial Pank and species in the mitochondrial milieu necessary for triggering mitochondrial-specific metabolic remodeling or for the activation of a specific program or cascade that leads to PKAN pathogenesis?

### Other mouse models

Transgenic mice overexpressing the *Nudt7* gene, which encodes a peroxisomal nudix hydrolase that mediates degradation of CoA, has also been used to study PKAN (38). DNA encoding the *Nudt7* gene linked to Syn promoter was delivered to mice *via* an adeno-associated virus to induce neuron-specific degradation of CoA (38). The overall effect was a reduction in whole brain CoA levels by 15% (38), though a survey of regions of the brain with the largest declines in CoA levels was not provided. The mice performed worse on tests of motor coordination compared with the syngeneic WT mice, despite similar grip strength. This indicates a neuronal deficit in motor function unrelated to muscle strength, which is similar to the motor disorder observed in PKAN. However, the overall pathology seen in these mice was mild compared with the clinical presentation seen in PKAN patients. This could be due to the fact that only 15% reduction of CoA levels in the brain of these mice was measured or to the existence of compensatory mechanisms that mitigate the effects of Nudt7 overexpression, perhaps through salvaging of acyl-P-PaSHs. In addition to providing another novel strategy for the study of PKAN, the model provides a proof of concept for gene therapy approaches based on adeno-associated virus delivery of genes invovled in CoA synthesis and utilization. This strategy could allow for delivery of a normal *PANK2* gene to targeted cells, thereby complementing the mutant allele in PKAN patients, an approach that has seen success in treatment of other rare diseases (39, 40).

## Modeling PKAN in nonmammalian hosts

### Zebrafish model

Models of PKAN in zebrafish have also identified key avenues for further elucidation of PKAN pathogenesis (20, 25). Attempts to create PKAN models in zebrafish have largely focused on generating *PANK2* hypomorphic mutants in tadpoles and adult fish. In one study, knockdown was achieved *via* injection of a splice-inhibiting morpholino, which resulted in disturbed brain morphology and hydrocephalus (20). There were also several vascular defects, including edema in the heart regions and caudal plexus, as well as hemorrhaging (25). In another study, overexpression of mutant human *PANK2* and mutant zebrafish *PANK2* mRNA was achieved in zebrafish embryos (25) and found to cause vascular and neurological defects and reduced locomotor behavior (25). Neurological defects are predictable in a model of PKAN, whereas vascular defects have not been described in any other model of PKAN. It is possible that these defects are specific to zebrafish. However, they might also indicate a previously unexplored link between *PANK2* activity and CoA homeostasis in vascular development. Importantly, overexpression of *PANK2* mutant forms appears to be associated with perturbation in CoA availability, irrespective of their catalytic activity (25). This mirrors the phenomenon seen in humans where some patients have WT levels of activity in the PANK2 enzyme but still develop symptoms associated with PKAN (41). Developing a better understanding of the possible alternative roles that the PANK2 enzyme or CoA might play in vascular development therefore remains critical to understanding the complete mechanism of PKAN pathogenesis.

### *Drosophila* model

Several attempts have been made to produce a viable PKAN model in *Drosophila* with varying degrees of success (1, 21, 23, 26, 42). In *Drosophila*, Pank activity is encoded by a single copy *fbl* gene, which is alternatively spliced into several different isoforms. Models targeting the *fbl* gene reconstituted various phenotypic features of human PKAN, including azoospermia, locomotor defects, and decreased life span (21). The longest form of *fbl*, fblL, was localized primarily to the mitochondria, whereas the shorter isoforms, fblS1 and fblS2, were found largely in the cytosol (21). Interestingly, whereas human *PANK3* transgene rescued some but not all the phenotypes associated with the loss of *fbl*, expression of the mitochondrial full-length fblL, or the human PANK2 harboring a mitochondrial targeting motif, resulted in full complementation of those phenotypes (21). This suggests that the mitochondrial localization of fbl, and PANK2, is critical for enzymatic activity and function. Surprisingly, expression of human *PANK4*, which lacks Pank activity, rescued *Drosophila* locomotor defects for the first 30 days and increased the average life span of the flies but did not restore male fertility. Whether this successful complementation is the result of altered CoA biosynthesis and/or compartmentalization remains to be elucidated.

### Yeast model

Modeling PKAN in the lower eukaryote *S. cerevisiae* could be particularly useful as the pathway for pantothenate utilization, and CoA biosynthesis is highly conserved between yeast and humans. The ease of handling of yeast and its amenability to genetic and cell biological analyses make it an ideal system to study the impact of CoA deficiency on cellular metabolism and to evaluate novel therapeutic strategies to restore normal CoA biosynthesis in cells with altered Pank activity. The yeast pantothenate kinase homolog, Cab1, is the sole PANK enzyme encoded by the yeast genome and is essential for cell viability (43). A Cab1 mutant carrying an amino acid substitution G351S is viable at 30 °C but not 37 °C and survives with only ∼7% of WT endogenous PANK activity (22). The mutant has been shown to exhibit defects in sterol biosynthesis leading to altered susceptibility to various classes of antifungal agents (22). A recent study showed that the Cab1^G315S^ mutant recapitulates the cellular defects found in cells isolated from PKAN patients (44). Colorimetric assays of iron content revealed significantly higher levels of intracellular iron despite decreased expression levels of key iron uptake genes in the iron regulon (44). Studying the mechanisms that cause this iron dyshomeostasis could help to clarify the role of excess iron in PKAN pathogenesis in humans. Moreover, yeast models of PKAN had a reduced oxygen consumption rate as well as NADH cytochrome *c* reductase and cytochrome *c* oxidase activities, indicating mitochondrial dysfunction (44). This same dysfunction is hypothesized to cause the deficits seen in PKAN (12). Further studies in yeast might help to identify biomarker of PKAN and validate theories of PKAN pathogenesis.

## Therapeutic strategies for PKAN

**Figure 4:**
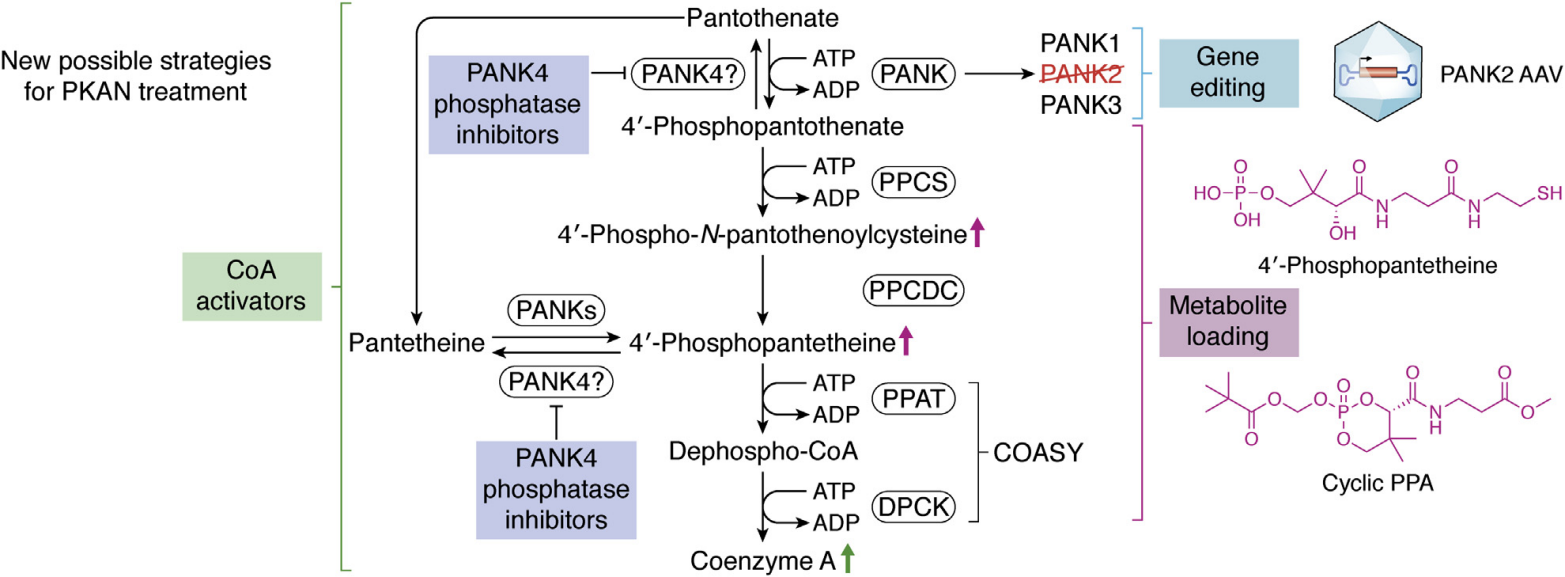
**Proposed alternative therapeutic strategies for PKAN.** Disease-altering therapeutics for PKAN have thus far focused largely on metabolite supplementation or stimulation of CoA synthesis *via* PANK activation. Gene therapies based on adeno-associated viral vectors or other novel delivery mechanisms as well as inhibitors that target enzymes, such as PPP89, that counteract the flow of production of CoA from pantothenate are promising new approaches. PKAN, pantothenate kinase–associated neurodegeneration.

**Figure 5:**
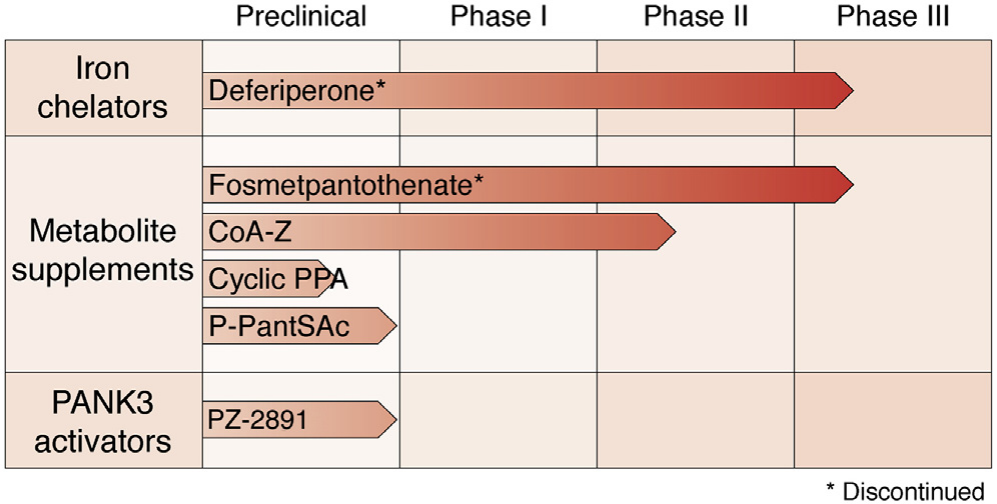
**Current stage of development for key PKAN therapeutics.** Some repurposed drugs focused on iron chelation (trial ID: NCT01741532) and metabolite supplementation (trial ID: NCT03041116) have completed phase III clinical trials in PKAN patients with little to no success. These drugs have been discontinued as treatments for PKAN. 4′-phosphopantotheine supplementation (Coa-Z trial ID: NCT04182763), acetyl-4′-phosphopantotheine supplementation, and PANK3 activation by BBP671 are promising new strategies for PKAN treatment at various stages of clinical evaluation. PKAN, pantothenate kinase–associated neurodegeneration.

There is currently no Food and Drug Administration–approved disease-modifying therapy available for PKAN. The current standard of care is geared toward managing individual symptoms (45). However, there are several promising treatments currently under active investigation (13, 14, 46, 47, 47). These treatments can be grouped under four broad approaches: iron chelation to treat brain iron deposits, metabolite supplementation to restore metabolic deficits in CoA pathway, PANK3 activation to restore CoA and phosphopantothenic acid levels, and gene therapy to introduce a functional copy of the *PANK2* gene (Fig. 4). Some of these therapies have not been successful, whereas others are at the preclinical or clinical stages of evaluation (Fig. 5).

It remains to be determined whether these treatment approaches could eliminate already accumulated cellular damages caused by years of severe disease progression or merely prevent worsening of symptoms. Moreover, recent studies have shown that PANK4 is a pseudokinase with an active phosphatase domain and can dephosphorylate P-PaSH and P-Pa (28). Should these biochemical data prove relevant to the regulation of the CoA pathway in cells, PANK4 activity could present another challenge to current metabolic-targeted therapies aimed at activating CoA biosynthesis (28).

### Iron chelators

The first major approach to the treatment of PKAN, iron chelation, focuses on treating the most prominent feature of PKAN, the characteristic iron deposits in the GP (1). Deferiprone (Ferriprox, ApoPharma), a well-known iron-chelating agent, was tested in phase II and phase III clinical trials (46). While patients did have significant reductions in iron deposits after treatment with the drug, the trials failed to reach their primary endpoint, which was a significant reduction in PKAN–activities of daily living score, a self-reported measure of disease severity (46). Importantly, even if treatment with Deferiperone alone is not effective in treating PKAN, iron-chelating drugs like Deferiperone might prove useful in combination with other treatment strategies for PKAN.

### Metabolite loading

The second major approach to PKAN treatment aimed to restore phosphopanthenate levels in PKAN cells. The first generation of drugs in this category focused on the delivery of exogenous P-Pa into affected cells. The largest challenge was making the drug both orally bioavailable and able to cross the blood–brain barrier. Prodrugs with improved membrane permeability were designed and showed some level of success in the cell models; one of which, Fosmetpantotenate, was further advanced to late-stage clinical trials (48). However, the drug failed to meet key primary and secondary endpoints in critical stage II clinical trial with 84 PKAN patients, and its development was subsequently discontinued (48). More recently, a cyclic phosphopantothenic acid prodrug, conceptually similar to Fosmetpantotenate, showed promising results in a human PANK2^*−/−*^ neuroblastoma cell line, increasing CoA levels (49). The prodrug has favorable physicochemical properties and increases phosphopantothenic acid in cells (49). The efficacy of this prodrug in mouse models of PKAN or in clinical trials remains to be evaluated.

Other efforts to treat PKAN include supplementation with CoA, pantethine, P-PaSH, and acetyl-P-PaSH (13, 50, 51, 52) to compensate for the reduced levels of the metabolic intermediates of CoA caused by altered Pank2 activity. 4′-Phoshphopantetheine was found to be the most effective of these compounds because it is both orally bioavailable and able to cross the blood–brain barrier (13). The efficacy of P-PaSH supplementation was validated in PKAN-derived fibroblasts, where expression levels of key enzymes in the CoA biosynthesis pathway were restored, and defects in iron, dopamine, and complex I activity were corrected (13). In mice lacking PANK2, the compound was found to enhance expression levels of specific biomarkers of PKAN (13). An ongoing phase II clinical trial for the use of P-PaSH (CoA-Z clinical trial no.: NCT04182763) in PKAN patients (initiated in December 2019) may shed light on the viability of this strategy for the treatment of PKAN. The acetyl-CoA precursor, S-acetyl-4′-phosphopantetheine (TM-1803) also showed promise in both *in vitro* and *in vivo* models (52). The compound, which was found to be stable in serum and showed no toxicity in human embryonic kidney 293 cells up to 500 μM (52), has been granted Orphan Drug Designation by the European Medicines Agency (53). S-acetyl-4′-phosphopantetheine has yet to advance to the clinical trial stage.

Overall, the *in vivo* efficacy data using mouse models of PKAN suggest that metabolite supplementation approaches could be an effective treatment to PKAN (13).

### Activation of CoA biosynthesis

Another approach under exploration for the treatment of PKAN involves the use of small-molecule activators of human PANK3. This approach aims to alleviate defects associated with mutations in *PANK2* by activating PANK3, an isoform of human PANK that is typically found in the cytosol or nucleus. A proof-of-concept preclinical study in mice showed that PZ-2891 was able to activate PANK3 by blocking an allosteric site necessary for feedback inhibition by acetyl-CoA (14). PZ-2891 was tested in mice with neuron-specific knockouts of *PANK1* and *PANK2*, where it was found to reduce weight loss and increase life span significantly (14). An activator-based approach, unlike metabolite supplementation and iron chelation, could alleviate toxicity resulting from excess cysteine in Pank2-deficient cells. However, there remains several unanswered questions about the promise of this approach. First, changes in CoA levels might not be important to PKAN pathogenesis as some patients express PANK2 enzymes with near WT levels of activity (*in vitro*) (54). If PKAN pathogenesis is linked to specific mitochondrially localized CoA pools, then it remains unknown whether activation of a nuclear enzyme or a cytosolic enzyme would be sufficient to restore the defect. Moreover, while PZ-2891 acts as an activator of CoA biosynthesis by altering feedback inhibition of human PANK3 by acetyl-CoA at concentrations up to 10 μM, the compound itself is a nanomolar inhibitor of human PANK3 and inhibits all other human PANK isoforms (14). This might lead to a narrow therapeutic window, creating difficulties with safe dosing levels. Despite these concerns, an activator-based strategy, which could be combined with iron chelators, might represent an alternative therapeutic strategy for the treatment of PKAN.

### PANK4 phosphatase activity and therapeutic implications

The role of PANK4 in PKAN has largely been ignored because of two substitutions in the catalytic domain of the amino terminus (Glu138Val and Arg207Trp) that are predicted to inactivate the kinase domain (28). While PANK4 shares homology with other human PANK enzymes, it is also uniquely endowed with a metal-dependent phosphatase domain DUF89 (27). Biochemical characterization of a purified recombinant moiety encompassing the PANK4 DUF89 domain was found to have phosphatase activity toward P-Pa, P-PaSH, and an even higher preference for an oxidized form of P-PaSH, S-sulfonate phosphopantetheine (27). While the phosphatase activity can be adaptive in some cases, in PKAN patients, this activity might further reduce the availability of 4′-phosphopanthenate and 4′-phosphopantetheine for CoA production, thus exacerbating CoA shortages in PKAN cells and resulting in more severe symptoms. The possible role of PANK4 in the exacerbation of the metabolic stresses related to PKAN could have major implications for therapies under consideration. PANK4 phosphatase activity might counteract the therapeutic benefits of small-molecule activators or metabolite supplementation with phosphorylated substrates, such as 4′-phosphopantetheine or CoA pathway precursors that are immediately phosphorylated by PANKs, including pantothenate and pantetheine. To compensate for this obstacle, therapeutic strategies relying on activators or metabolite supplementation may require the use of maximum tolerable doses of these metabolites to achieve efficacy. Another solution could be to identify specific inhibitors of the PANK4 phosphatase activity to be used alone or in combination with small-molecule activators or metabolites.

### Gene therapies

Monogenic disorders are often ideal candidates for gene therapy including gene editing. Gene therapy can be achieved by the introduction of a single functional copy of *PANK2*, a relatively small gene (∼5 kb and its complementary DNA is <2 kb in length) to fully compensate for any metabolic defects and treat PKAN. A variety of vectors have been used in recent years to introduce functional proteins to the brain, including in efforts to treat Alzheimer’s disease (55). Moreover, in 2018, the Food and Drug Administration–approved Luxturna (voretigene neparvovec-rzyl), a single dose gene therapy delivered by subretinal injection to treat a rare form of retinal dystrophy (40). Thus, a functional gene therapy treatment for PKAN might be a viable option in the near future. However, application of a gene-based therapy for PKAN remains challenging for several reasons. The first complication is that any vector carrying the gene would have to be delivered across the blood–brain barrier. One way to solve this problem could be to take advantage of recent advances in adeno-associated vectors that have produced a serotype capable of entering the brain after intravascular injection (56, 57). Alternatively, gene therapies could be delivered *via* stereotactic injection after a craniotomy. The second key issue for gene therapy would be inducing widespread stable gene expression over a long period, without inducing inflammation. Once again, adeno-associated vectors have been shown to induce long-lasting expression with transduction of quiescent cells and a low risk of immune response (58). Finally, a recent study using transgenic mice overexpressing human PANK2 showed that CoA levels were substantially elevated in skeletal muscle. These mice were smaller, had less skeletal muscle mass, and displayed significantly impaired exercise tolerance and grip strength (59). These findings suggest that gene therapy–based approaches should be first validated in animal models and must rely on detailed investigations of the impact of overexpressing Pank2 or other genes in the CoA biosynthesis pathway on global metabolism and physiology.

## Conclusions and future directions

Herein, we analyzed data from models of PKAN to understand the etiological mechanisms underlying the disease and translate this knowledge to possible therapies for this disorder. Although CoA levels in some patient-derived cell lines appear to be no different from controls, various defects associated with PKAN are likely to be caused by reduced levels of CoA or downstream metabolites in the brain (9, 13). These might go undetected in cell lines because the defects are limited to critical subcellular pools such as those required to activate mtACPs (13). The data also suggest that iron accumulation, the pathognomonic hallmark of PKAN, might be caused by a primary metabolic defect in CoA rather than a primary driver of disease progression. Nevertheless, further studies into the mechanisms of iron accumulation are important for understanding how iron dyshomeostasis affects neurodegeneration in other diseases like Parkinson’s disease and Alzheimer’s disease. Large-scale MultiOMIC studies in yeast and *pank2*^*−/−*^ mouse models could aid our understanding of these mechanisms and identify specific biomarkers of PKAN. Further research into the global effects of PANK2 mutation in other organs is also important to gaining a comprehensive understanding of the disease. Driven by advances in understanding the complex etiologies of PKAN, new treatment options for this disease are currently under active development. Particularly promising approaches include activation of CoA biosynthesis by PANK activators and gene therapies.

## Conflict of interest

C. B. M. is the founder of Virtus Therapeutics whose focus is to develop new therapies for PKAN. The other authors declare no conflicts of interest with the contents of this article.
